# Supplementary material for: Large Extracellular Vesicle Characterization and Association with Circulating Tumor Cells in Metastatic Castrate Resistant Prostate Cancer
Source: Cancers (Basel). 2021 Mar 2;13(5):1056. doi: 10.3390/cancers13051056 (PMC7958848; doi:10.3390/cancers13051056)
Supplement: Supplementary file 1 [file cancers-13-01056-s001.pdf]

cancers

MDPI

Supplementary Materials

Large Extracellular Vesicle Characterization and Association with Circulating Tumor Cells in Metastatic Castrate Resistant Prostate Cancer

Anna S. Gerdtsson, Sonia M. Setayesh, Paymaneh D. Malihi, Carmen Ruiz, Anders Carlsson, Rafael Nevarez, Nicholas Matsumoto, Erik Gerdtsson, Amado Zurita, Christopher Logothetis, Paul G. Corn, Ana M. Aparicio, James Hicks and Peter Kuhn

Table S1: Antibodies used for IMC.

Index	Metal	Marker	Rationale	Vendor	Clone	Isotype	Dilution
1	Ir193	DNA		Fluidigm			
2	In115	Membrane		Fluidigm			
3	Y89	CD45	WBC marker / CTC neg. / Normalization	Fluidigm catalog	HI30	Mouse IgG1	200
4	Pr141	EpCAM	Epithelial / CTC marker	Fluidigm catalog	9C4	Mouse IgG2b	100
5	Nd142	CD9	General EV (tetraspanin) marker	Lsbio*	LS-C416960	Rabbit IgG	100
6	Nd143	AR-N	PC marker	Cell signaling*	D6F11	Rabbit IgG	100
7	Nd145	CD31	Endothelial marker	Fluidigm	WM59	Mouse IgG1	100
8	Nd146	BCL-2	Proliferation marker	Fluidigm	EPR17509	Rabbit IgG	100
9	Sm147	B-catenin	wnt/EMT, PC progression marker	Fluidigm	D10A8	Rabbit IgG	100
10	Nd148	PD-L1	Checkpoint regulation marker	Fluidigm	SP142	Rabbit IgG	100
11	Sm149	cav-1	PC LO marker / PC progression marker	Novus*	7C8	Mouse IgG2b	50
12	Nd150	Sox2	PC progression/LN metastasis marker	Fluidim	O30-678	Mouse IgG1	100
13	Eu151	ATP5B	PC LO /angiogenesis, endocytosis, migration marker	Novus*	NBP1-90816	Rabbit IgG	25
14	Sm152	TWIST-1	EMT marker			Rabbit IgG	50
15	Eu153	ERG	marker of TMPRSS2 fusion, AR independence in PC	Abcam*	EPR3864 (2)	Rabbit IgG	25
16	Sm154	Vimentin	EMT marker / discriminate from endothelial cells	Fluidigm	D21H3	Rabbit IgG	100
17	Gd155	HSPD1	PC LO marker / cell proliferation, PC progression	Novus*	NBP1-77397	Rabbit IgG	50
18	Gd156	p-p38	General NF-kB signaling / PC survival, metastasis	Fluidigm	D3F9	Rabbit IgG	100
19	Gd158	E-cadherin	Epithelial marker / EMT	Fluidigm	24E10	Rabbit IgG	100
20	Gd160	PSA	PC marker	Fluidigm custom	TD11B3-4	Mouse IgG1	100
21	Dy161	LGR5	Stem cell marker	Fluidigm	4D11F8	Rat	50
22	Dy162	HSPA5	PC LO marker / proliferation / glutamine metabolism	Novus*	NBP2-16749	Rabbit IgG	50
23	Dy163	AR-V7	PC marker	Fluidigm custom	EPR15656	Mouse IgG2a	100
24	Dy164	AR-C	PC marker	Abcam*	SP242	Rabbit IgG	100
25	Ho165	PD1	Checkpoint / immune marker	Fluidigm	EPR4877(2)	Rabbit IgG	100
26	Er166	CD24	Tumor stem cell marker	Fluidigm	ML5	Mouse IgG2a	100
27	Er167	pERK1/2	MAPK signaling / PC progression marker	Fluidigm	D.13.14.4E	Rabbit IgG	50
28	Er168	Ki-67	Proliferation marker	Abcam*	ab15580	Rabbit IgG	50
29	Tm169	PSMA	PC marker	Novus*	460420	Mouse IgG2a	100
30	Er170	Survivin	Proliferation / observed in PC exosomes	Novus*	1277A	Rabbit IgG	50
31	Yb171	CD44	Tumor stem cell marker	Fluidigm	IM7	Rat IgG2b	400
32	Yb172	Caspase-3 (cleaved)	Apoptosis marker	Fluidigm	5A1E	Rabbit IgG	100
33	Yb173	CD59	Prostatosome marker	Fluidigm	p828 (H19)	Mouse IgG2a	50
34	Yb174	CK8/18	Epithelial / CTC, LO marker	Fluidigm	C51	Mouse IgG1	200
35	Lu175	CXCR4	Migration, invasion, PC metastasis	Fluidigm	NBP1-77067	Rabbit IgG	50

*In-house conjugated.

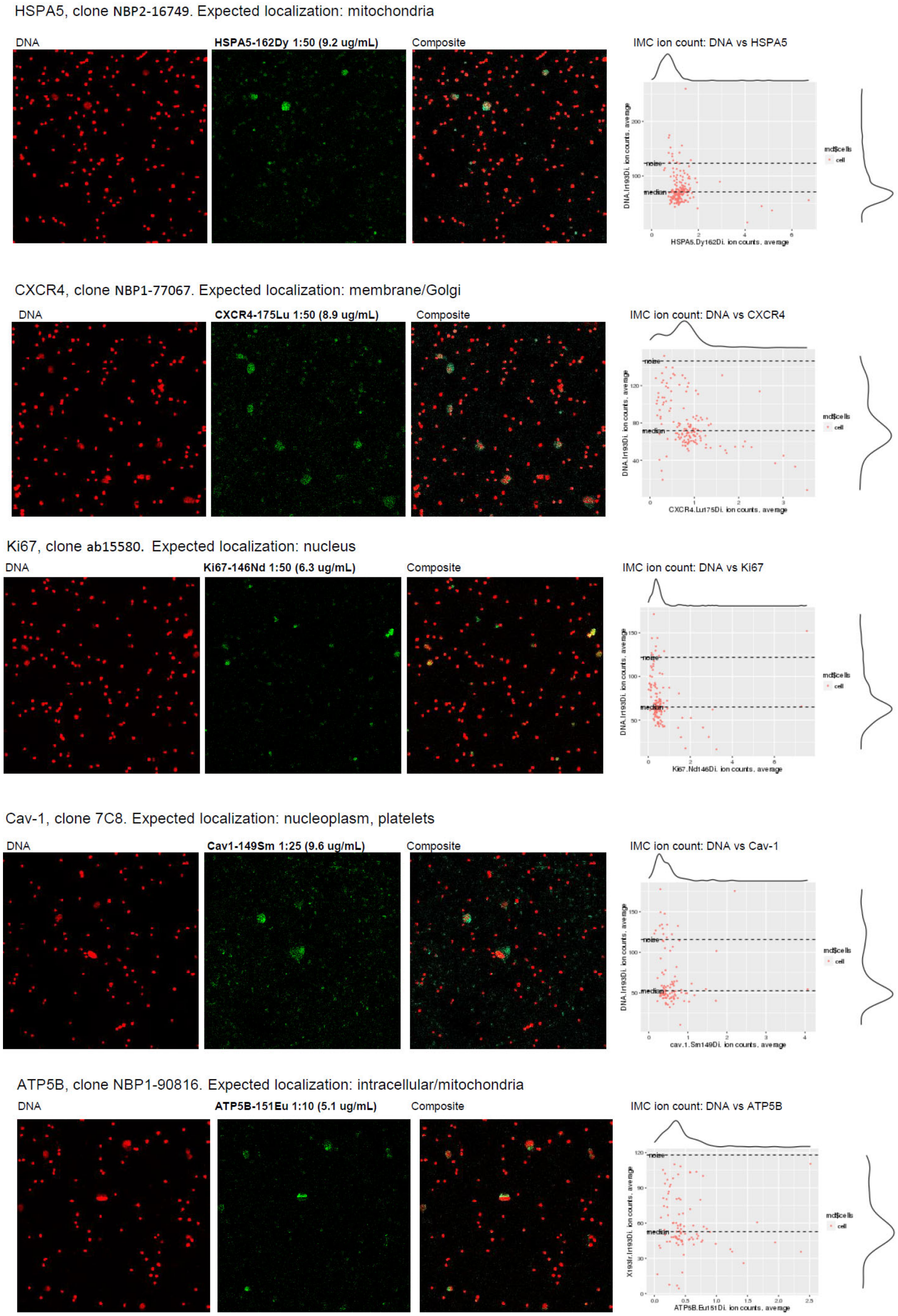

Figure S1. Staining of in-house conjugated IMC antibodies using PC3 cells spiked at 1:100 ratio (tumor cells: WBC) and processed by the HD-SCA protocol c.

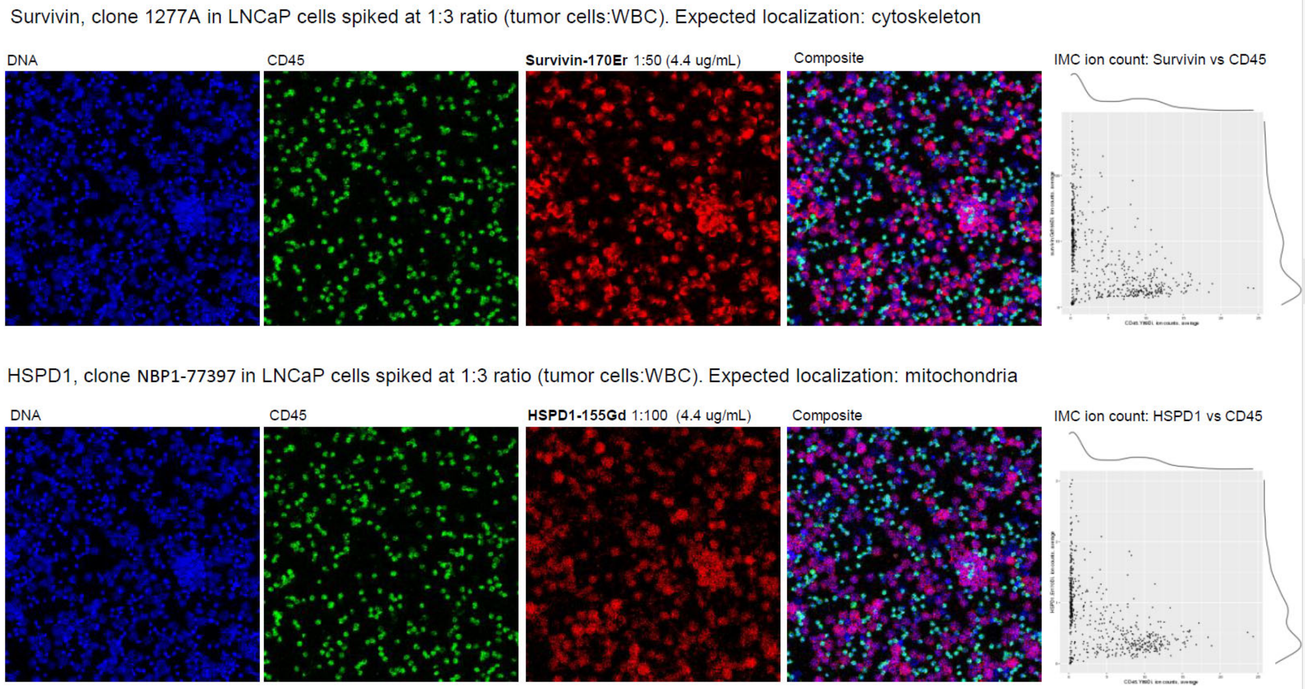

Figure S2. Staining of in-house conjugated IMC antibodies using LNCaP cells spiked at 1:3 ratio (tumor cells: WBC) and processed by the HD-SCA protocol.

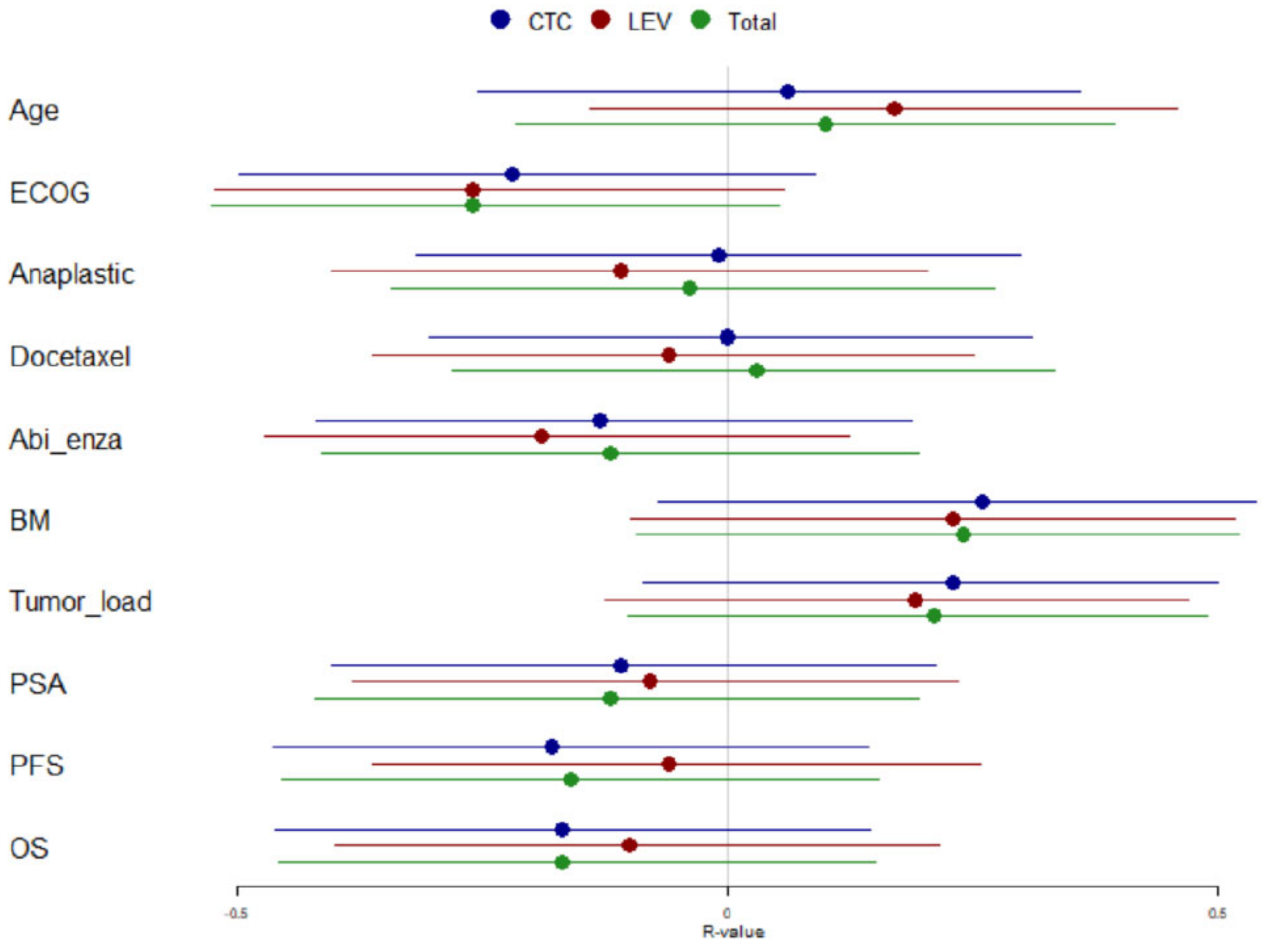

Figure S3. Correlation of CTC and LEV enumeration to clinical parameters. Pearson R-value with 95% confidence intervals are shown.

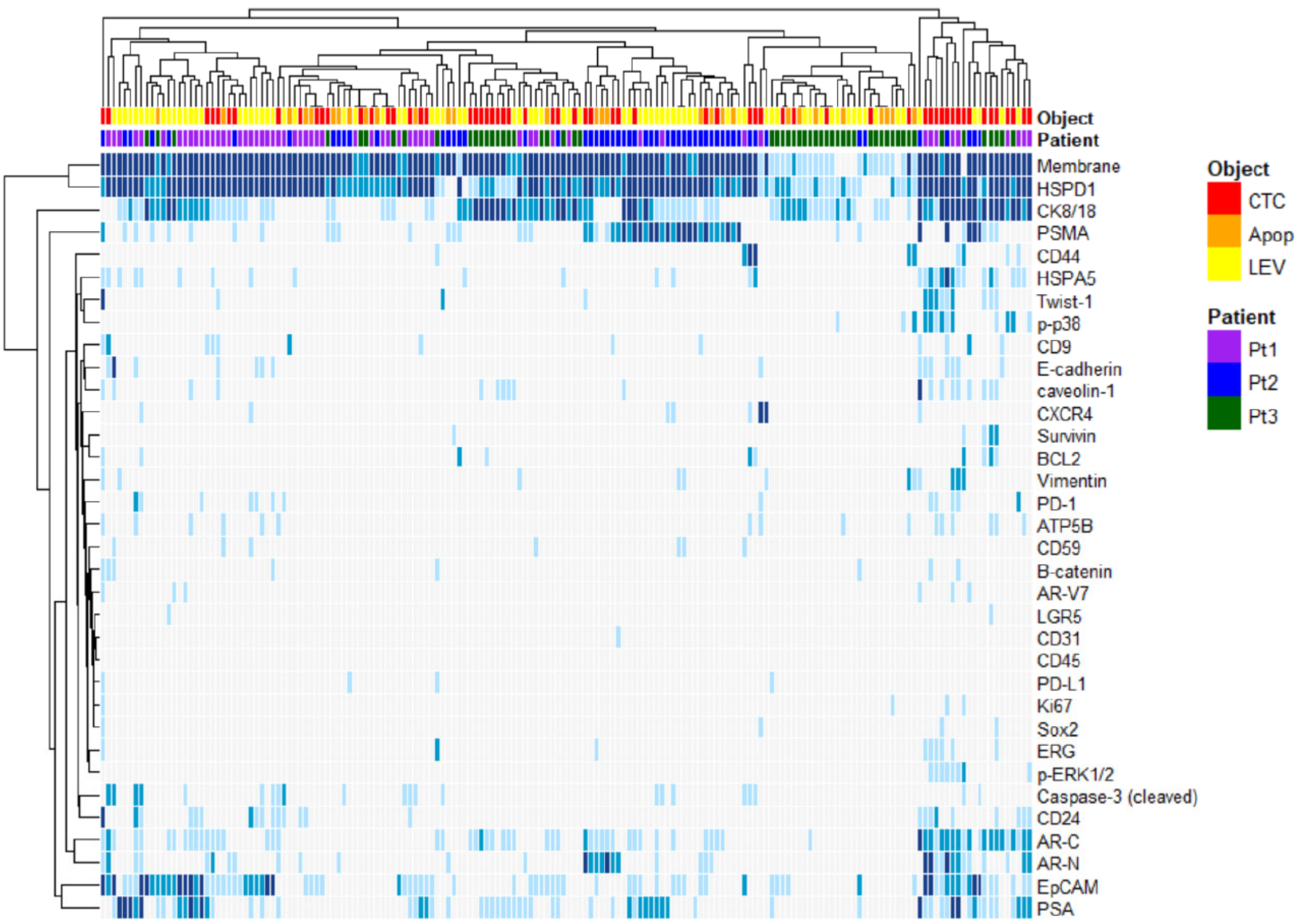

Figure 4. Clustering of LOs, CTCs, and Apoptoticsbased on IMC data from 3 patients. When excluding DNA, objects were poorly clustered by category.

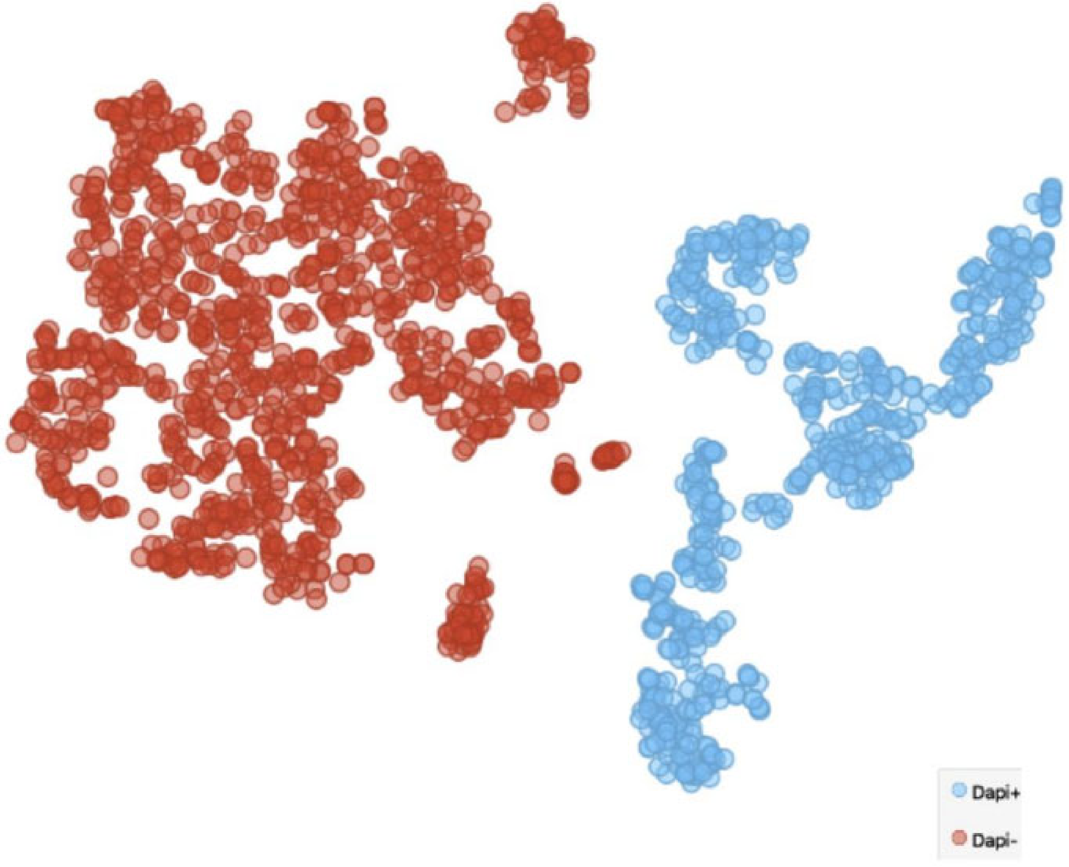

Figure 5. Clustering of LEVs (red) and CTCs (blue) by Tsne based on all morphometric parameters (n = 444) collected in the HD-SCA report system, including measurements based on DAPI.
